# Association of *VDR* gene *Apal* polymorphism with obesity in Iranian population

**DOI:** 10.7705/biomedica.5898

**Published:** 2021-12-15

**Authors:** Farzad Rashidi, Maryam Ostadsharif

**Affiliations:** 1 Departamento de Immunología, Escuela de Medicina, Universidad Complutense, Madrid, España Universidad Complutense de Madrid Departamento de Immunología Universidad Complutense Madrid Spain; 2 Young Researchers and Elite Club, Isfahan (Khorasgan) Branch, Islamic Azad University, Isfahan, Iran Islamic Azad University Islamic Azad University Isfahan Iran; 3 Department of Medical Basic Sciences, Isfahan (Khorasgan) Branch, Islamic Azad University, Isfahan, Iran Islamic Azad University Department of Medical Basic Sciences Islamic Azad University Isfahan Iran; 4 Department of Medical Biotechnology, Isfahan (Khorasgan) Branch, Islamic Azad University, Isfahan, Iran Islamic Azad University Department of Medical Biotechnology Islamic Azad University Isfahan Iran

**Keywords:** Obesity/genetic, vitamin D, polymorphism, genetic, body mass index, Iran, obesidad/genética, vitamina D, polimorfismo genético, índice de masa corporal, Irán

## Abstract

**Introduction:**

Identifying obesity risk factors as a health problem facing communities is crucial given its complexity. The vitamin D receptor gene has been reported as a possible cause of this disease.

**Objective:**

To study the association of the *VDR* gene *Apa*I, *Bsm*I, and *Taq*I polymorphisms with obesity in an Iranian population.

**Material and methods:**

We analyzed the genotypes of 348 obese (BMI≥30 kg/m^2^) and 320 non-obese people (BMI: 18.5-24.9 kg/m^2^) using PCR-RFLP. We measured FBS, TG, total cholesterol, and HDL and LDL cholesterol levels in an automatic biochemical analyzer.

**Results:**

We found significantly higher BMI, FBS, and TG levels in the obese group compared to the control. In the obese individuals, the frequency of genotype AA was 47.1% and that of the combined Aa+aa genotype, 52.9% while in the control group they were 30% and 70%, respectively (p=0.024, 95% confidence interval (CI)=1.100-3.933, odds ratio (OR)=2.08). A and a alleles frequencies for the ApaI polymorphism were statistically significant in the two groups (allele A vs. a; p=0.017). No significant relationship was observed between *Taq*I genotypes and alleles in the control and obese subjects.

**Conclusion:**

We found that VDR *Apa*I (rs7975232 C/A) polymorphism appeared to be a risk factor for obesity. Especially, the A allele and the AA genotype in *Apa*I were associated with the obesity phenotypes.

## Resumen

**Introducción.:**

La determinación de los factores de riesgo de la obesidad en la población iraní como problema de salud de la comunidad es crucial dada su complejidad. El gen receptor de la vitamina D (*VDR*) se ha mencionado como posible causante de dicha enfermedad.

**Objetivo.:**

El objetivo del estudio fue investigar la asociación de los polimorfismos *Apa*l, *Bsm*l y *Taq*l, con el gen *VDR* y la obesidad en una población iraní.

**Materiales y métodos.:**

Se analizaron genotipos de 348 individuos obesos (BMI>30 kg/m^2^) y 320 no obesos (BMI: 18,5-24,9 kg/mf) mediante reacción en cadena de la polimerasa y polimorfismos de longitud de fragmentos de restricción (PCR-RFLP). Para medir los niveles de glucemia en ayunas, tiroglobulina (TG), colesterol total, colesterol HDL y colesterol LDL, se utilizó un analizador bioquímico automático.

**Resultados.:**

Los índices de masa corporal, glucemia en ayunas y TG fueron significantemente más elevados en el grupo de los obesos que en el de control. En los individuos obesos, la incidencia del genotipo AA fue de 47,1 % y la del genotipo combinado Aa+aaa fue de 52,9 %, en tanto que en el grupo de control estas cifras fueron, respectivamente, de 30 y 70 % (p=0,024; IC_95%_ 1,100-3,933; la razón de probabilidades (OR) fue de 2,08. La frecuencia de los alelos “A” y “a” para el polimorfismo *Apa*l en ambos grupos fue estadísticamente significativa (alelo A *Vs*. A; p=0,017). No se observó ninguna relación significativa entre los genotipos *Taq*l y los alelos en los sujetos obesos y, tampoco, en los controles.

**Conclusión.:**

El polimorfismo *Apa*l del gen *VDR* (rs 7975232C/A) sería un factor de riesgo para la obesidad. El alelo y el genotipo AA en dicho polimorfismo se asociaron con los fenotipos de obesidad.

Obesity is a complex disease influenced by environmental and genetic factors, which consists of the excessive accumulation of fats in adipose tissues as a result of an imbalance between energy consumption and intake. In the past 50 years, unhealthy habits in lifestyle have resulted in the so-called “obesogenic” environment, i.e., the intake of easily accessible energy-dense foods coupled with decreased physical activities. Researchers have suggested that genetic factors play a key role in regulating body weight ^1^. Obesity can cause many disorders, including cancer, cardiovascular disease, impaired glucose tolerance, hypertension, type 2 diabetes, sleep apnea, osteoarthritis, and gallbladder and liver disorders ^2^ The body mass index (BMI) has been commonly used as a surrogate marker of excessive body fats in the absence of accurate yet simple techniques for measuring them ^3^. Identifying the genetic factors contributing to the risk of obesity may help broaden the basic biological knowledge about the energy imbalance and determine the pathways and molecules that may be targeted for therapeutic purposes in humans.

Although vitamin D deficiency is a proven risk factor for obesity ^4^^-^^6^, the exact relationship between vitamin D status and obesity remains unclear. Vitamin D function is induced by the attachment of 1,25-dihydroxyvitamin D3, one of vitamin D active forms, to VDR as a nuclear receptor and a product of the *VDR* gene locus on chr12q13.1. A nuclear receptor acts as a ligandinducible transcription factor. Some polymorphisms reported in the *VDR* gene are associated with certain diseases and phenotypes ^7^ Several *VDR* gene polymorphisms, including *Taq*I, *Bsm*I, and *Apa*I, are located near the 3’ un-translated region (UTR). In the human VDR, the *Fok*I translation-start site polymorphism was found in the 5’ UTR.

In Iran, the prevalence of overweight/obesity is 63.6% in adults while that of abdominal obesity is 75.2%, and it is higher in women (32.2%) than in men (15.1%) ^8^. Mirzazadeh, *et al*., have reported an obesity prevalence of 13.7% in men and 27.3% in women ^9^. Frequently, *VDR* gene polymorphisms have been found to be related to obesity, although these findings differ depending on the population ^10^^-^^12^. Considering them as risk factors for obesity, we studied three of these polymorphisms: *Taq*I, *Apa*I, and *Bsm*I, and their relationships with serum factors in an obese Iranian population.

## Materials and methods

### Data collection and participants

We conducted a case-control study in the private nutrition clinic of Abolabbas, a charity institution in Khorasgan, Isfahan, Iran, from October, 2014, to March, 2015. Multiple clinical evaluations were performed and a questionnaire was completed by participants. The parameters recorded included weight, height, and BMI calculated by the accurate measurement of height (meters) and weight (kg) under the supervision of a nutritionist. According to the BMI classification proposed by the World Health Organization (WHO), 320 subjects were categorized as healthy controls with normal weight (BMI: 18.5-24.9 kg/m^2^) and no chronic diseases while 348 participants were considered obese individuals (BMI≥30 kg/m^2^) ^13^; their ages ranged from 25 to 80 years. Pregnant or breastfeeding women were excluded, as well as those with a family history of obesity, severe psychological disorders, or other serious diseases, and those consuming metformin, vitamin D, calcium supplements, or cholesterol-lowering medications.

We used biochemical kits to evaluate fasting blood sugar (FBS) (Biorexfars, Iran), triglycerides (TG) (ParsAzmun, Iran), total cholesterol (ParsAzmun, Iran), high-density lipoprotein cholesterol (HDL cholesterol) (ParsAzmun, Iran), and low-density lipoprotein cholesterol (LDL cholesterol) (calculated parameter) after eight hours of nocturnal fasting. The results of tests related to blood biochemical parameters were evaluated by an internist and a nutritionist.

All the participants signed written informed consent forms before sampling and data collection. The Research Committee of the Isfahan (Khorasgan) Branch, Islamic Azad University, Isfahan, Iran approved the present study.

### Genotyping

Blood samples were collected in EDTA tubes and DNA was extracted from peripheral blood leukocytes through standard salting-out. We used the PCR- RFLP technique with forward and reverse primers to amplify and analyze the genomic DNA of VDR genotype *Taq*I, *Apa*I, and *Bsm*I polymorphisms following the protocol of a previous study (14). Allelic nomenclatures of dominant (ABT) alleles are based on endonuclease success over its *Taq*I, *Apa*I, and *Bsm*I restriction sites. Recessive (abt) alleles were therefore utilized when these endonucleases failed to cut their corresponding DNA molecules.

### Data analyses

We analyzed the data using the SPSS-20.0™ software. We examined the Hardy-Weinberg genotype equilibrium in the control group using the chi- squared test. The descriptive data were expressed in means and standard deviations, as well as allele and genotype frequency. The Kolmogorov- Smirnov test was used to analyze the normality of distribution for each variable. To compare groups we used the chi-square test for qualitative data and for quantitative data, independent samples T-test. Odds ratios (OR) were calculated using logistic regression with 95% confidence intervals (CI). A p-value less than 0.05 was considered statistically significant.

## Results

The characteristics of the obese and control subjects and the details of the control group (mean age: 50.02 ± 12.47 years) and the obese participants (mean age: 36.30 ± 10.19 years) are shown in table 1. There were significantly more women in the obese group than in the control group. FBS, TG, and BMI were also significantly higher among the obese subjects compared to the controls. There were no significant differences between the two groups in terms of smoking status, alcohol consumption, HDL cholesterol, LDL cholesterol, total cholesterol, and heart disease; however, there were significantly more subjects who practiced physical activity in the control group than in the obese group (p=0.010).

**Table 1:** Anthropometric and biochemical characteristics of study subjects

Variable		Normal weight (Control group n=80)	Obese (Case group n=87)	p-value
Sex; n (%)	Male	Sex; n (%)	Sex; n (%)	0.002^a^
	Female			
Smoking status; n (%)	Yes	Smoking status; n (%)	Smoking status; n (%)	0.342^a^
	No			
Physical activity; n (%)	Yes	Physical activity; n (%)	Physical activity; n (%)	0.010^a^
	No			
Alcohol intake; n (%)	Yes	Alcohol intake; n (%)	Alcohol intake; n (%)	0.428^b^
	No			
Heart disease status; n (%)	Yes	Heart disease status; n (%)	Heart disease status; n (%)	1.00^b^
	No			
Age (year)	Mean ± SD	Age (year)	Age (year)	<0.001^c^
BMI (kg/m2)	Mean ± SD	BMI (kg/m2)	BMI (kg/m2)	<0.001^d^
FBS (mg/dl)	Mean ± SD	FBS (mg/dl)	FBS (mg/dl)	<0.001^c^
LDL cholesterol (mg/dl)	Mean ± SD	LDL cholesterol (mg/dl)	LDL cholesterol (mg/dl)	0.244^c^
HDL cholesterol (mg/dl)	Mean ± SD	HDL cholesterol (mg/dl)	HDL cholesterol (mg/dl)	0.132^c^
Triglycerides (mg/dl)	Mean ± SD	Triglycerides (mg/dl)	Triglycerides (mg/dl)	<0.001^d^
Total cholesterol (mg/dl)	Mean ± SD	Total cholesterol (mg/dl)	Total cholesterol (mg/dl)	0.071^d^

p-values from a: Chi square test; b: Fisher exact test; c: Mann-Whitney test; d: independent t study

BMI: body mass index; FBS: fasting blood sugar; LDL: low-density lipoprotein; HDL: high-density lipoprotein

The Hardy-Weinberg equilibrium was observed in the *Taq*I and *Apa*I genotypes distributions in the control group (p=0.086 and 0.262, respectively) (table 2). A significant deviation was, however, observed in the Hardy- Weinberg equilibrium of the *Bsm*I polymorphism in the controls (p<0.001).

**Table 2:** Hardy-Weinberg equilibrium in the control group

Genotype		Observed	Expected	χ^2^	p-value
*Apal*	aa	12	14.5	1.256	0.262
	Aa	44	39.1		
	AA	24	26.5		
*Taql*	tt	8	11.6	2.957	0.086
	Tt	45	377		
	TT	27	30.6		
*Bsml*	bb	4	171	34.778	<0.001
	Bb	66	39.8		
	BB	10	23.1		

### VDR polymorphisms and obesity risk

Table 3 shows the allelic and genotypic frequencies of VDR *Apa*I and *Taq*I polymorphisms in both groups. *Apa*I and *Taq*I genotypes were expressed as recessive homozygous (aa, tt), dominant homozygous (AA, TT), and heterozygous (Aa, Tt). The frequency of genotype AA was 47.1% and that of the combined genotype Aa+aa, 52.9% in the obese group, figures that corresponded to 30% and 70% in the controls, were respectively (p=0.024, OR=2.08, 95%CI=1.100-3.933). Compared to genotype aa, Aa was found as a potential risk factor for obesity (p=0.029, OR=3.417, 95%CI=1.135-10.283). The frequencies of A and a alleles in the ApaI polymorphism were statistically significant in the two groups (allele A vs. a, p=0.017). As shown in table 3, there was no significant association between *Taq*I genotypes and alleles in neither group.

**Table 3:** *Apal* and *Taql* polymorphisms and obesity risk

Genotype	Obese (%)	Control (%)	p-value	OR	95%CI
*Apal*					
aa	6 (6.9)	12 (15.0)		1	
Aa	40 (46.0)	44 (55.0)	0.029	3.417	1.135-10.283
AA	41 (47.1)	24 (30.0)	0.273	1.818	0.624-5.298
Aa+aa	46 (52.9)	56 (70.0)		1	
AA	41 (47.1)	24 (30.0)	0.024	2.08	1.100-3.933
A	52 (29.9)	68 (42.5)		1	
A	122 (70.1)	92 (575)	0.017	1.734	1.104-2.723
*Taql*					
tt	8(9.2)	8(10.0)		1	
Tt	42(48.3)	45(56.3)	0.574	1.370	0.457-4.110
TT	37(42.5)	27(33.8)	0.899	0.933	0.321-2.711
Tt+tt	50 (57.5)	53 (66.3)		1	
TT	37(42.5)	27(33.8)	0.245	1.453	0.775-2.724
t	58 (33.3)	61 (38.1)		1	
T	116 (66.7)	99 (61.9)	0.361	1.232	0.787-1.930

P-value, OR and 95%CI based on logistic regression

### Apal genotypes associations with biochemical parameters

In table 4 we present the distribution of clinical variables by genotypes in the obese and control groups. We combined genotypes Aa and aa given the limited frequency of aa genotype in the VDR ApaI polymorphism. Moreover, 86.2% of the AA genotype and 70.6% of the Aa + aa group corresponded to women. According to the Chi-squared test, the two groups were significantly different in terms of gender distribution and the vast majority of the participants with genotype AA were female (p= 0.020).

**Table 4:** *Apal* polymorphism and obesity components

Variable		***Apal* AA**	***Apal* Aa+aa**	p-value
Gender, n (%)	Male	9 (13.8)	30 (29.4)	0.020^a^
	Female	56 (86.2)	72 (70.6)	
Heart disease, n (%)	Yes	3 (4.6)	1 (1.0)	0.300^b^
	No	62 (95.4)	101 (99.0)	
BMI (kg/m2)	Mean ± SD	31.56 ± 7.72	28.55 ± 6.62	0.022^c^
FBS (mg/dl)	Mean ± SD	114.61 ± 32.43	103.69 ± 29.41	0.003^c^
LDL cholesterol (mg/dl)	Mean ± SD	10771 ± 37.16	109.74 ± 31.34	0.718^d^
HDL cholesterol (mg/dl)	Mean ± SD	46.25 ± 12.59	45.33 ± 10.65	0.627^d^
Triglycerides (mg/dl)	Mean ± SD	162.98 ± 86.38	149.97 ± 82.56	0.242^c^
Total cholesterol (mg/dl)	Mean ± SD	184.29 ± 45.25	185.82 ± 43.65	0.832^d^

P-value from a: Chi square test; b: Fisher exact test; c: Mann-Whitney test; d: independent t study

BMI: body mass index; FBS: fasting blood sugar; LDL: low-density lipoprotein; HDL: high- density lipoprotein

As for the *Apa*I variant, BMI (p=0.022) and FBS (p=0.003) were higher in the subjects carrying the AA genotype compared to those with the Aa+aa genotype. Other parameters such as heart disease, LDL cholesterol, HDL cholesterol, TG, and total cholesterol were not associated with the AA and Aa+aa genotypes.

## Discussion

A complex feedback mechanism mediated by receptors, enzymes, and hormones, regulates 1,25(OH)_2_D. With a key function in bone and calcium homeostasis, 1,25(OH)_2_D/VDR signaling can regulate proliferation, differentiation, and various cellular responses in the cardiovascular and immune systems ^15^^,^^16^. The evidence suggests that VDR polymorphisms and vitamin D deficiency can increase the risk of osteoporosis, calcium stones, diabetes, and prostate cancer ^17^ and the first may affect development and growth processes.

A large body of literature has been devoted to four common singlenucleotide polymorphisms in the *VDR* gene: *Taq*I C>T (rs731236), *Apa*I C>A (rs7975232), *Fok*I T>C (rs10735810), and *Bsm*I A>G (rs1544410), whose relationships with different human diseases and traits have been explored ^18^. The single-nucleotide polymorphisms near the 3’ UTR include *Apa*I, *Taq*I, and *Bsm*I. Despite being non-functional, they are linked with a poly (A) microsatellite repeat in the 3’ UTR, which can affect the stability of the *VDR* mRNA, the efficiency of protein translation, and the modulation of gene expression while the protein expression can be affected by altered intronic sequences ^7^^,^^19^.

Our findings suggested an association between the *VDR Apa*I polymorphism (rs7975232 C/A) and the susceptibility to obesity while the A allele and the AA genotype in *Apa*I were associated with obesity phenotypes. The relationships of the *VDR* gene polymorphisms with the anthropometric and biochemical features of obesity were evident in the higher serum levels of FBS and BMI in genotype AA carriers.

Identifying the risk factors and genetic causes of obesity is crucial given its significant frequency in developing and developed countries. Risk factors are different in many populations and several *VDR* gene variants appear to affect obesity differently. A few studies suggest relationships between VDR polymorphisms and obesity ^11^^,^^20^^,^^21^. For example, Ferrarezi, *et al*., found that *Bsm*I polymorphism was related to the height in a cohort of obese adolescents and children but *Taq*I and *Apa*I were not significantly associated with obese adolescents and children ^22^. In 2018, Correa-Rodríguez, *et al*., found that *VDR* genetic variants did not contribute to obesity phenotypes in a population of Caucasian young adults ^23^.

A study in Saudi Arabia reported vitamin D deficiency, VDR *Bsm*I, and *Taq*1 genotypes as risk factors for obesity ^24^. In 2014, another study in Saudi Arabia by Al-Daghri, *et al*., revealed that polymorphisms affecting the vitamin D/VDR axis played a role in obesity in terms of the inflammation possibly caused by changes in microbial translocation and gut permeability. All the polymorphisms, namely *Taq*I, *Apa*I, and *Bsm*I, were found to relate to obesity ^20^. In 2015, Ostadsharif, *et al*., obtained the frequency of alleles F and f in obese and healthy groups. The difference between the two alleles in the control and obese groups was significant (p=0.005) and the individuals with the FF genotype in the control group had lower fasting blood sugar levels compared to the other genotypes ^25^. In contrast, a study in Poland reported no statistically significant differences in BMI, nor in the weight or height, for four VDR genotypes, i.e. *Taq*I, *Apa*I, *Bsm*I, and *Fok*I ^26^.

The study of *Apa*I and *Taq*I to evaluate VDR polymorphisms in nephropathic and non-nephropathic patients with type 2 diabetes conducted by Iranian researchers did not show relationships between the *Apa*I polymorphism and the nephropathic and non-nephropathic diabetic patients, although it showed significant differences in VDR gene *Taq*I genotypes of diabetic patients with and without nephropathy ^27^.

The contribution of *VDR* gene polymorphisms to susceptibility to neurodegenerative diseases and conditions associated with calcium metabolism has been frequently reported in the literature. In Japan, the lumbar spine BMD of the common genotype AA was lower by 9.3% than in genotype aa in premenopausal women ^28^. In Greece, no heritability patterns were reported for the relation of genotype AA and low BMD ^29^.

In Iran, Meamar, *et al*., evidenced that *Fok*I f and *Apa*I a alleles significantly increase the risk of Parkinson’s disease as was the case of *Apa*I heterozygous genotype Aa compared to the AA homozygous ^14^.

In the present study, we found higher BMI and FBS levels in those subjects with genotype AA than in those with genotype Aa+aa. In a metaanalysis, the authors found significant relations between the VDR gene *Taq*I, *Bsm*I, *Apa*I, and *Fok*I polymorphisms and the risk of type 2 diabetes; *Fok*I polymorphism was a risk factor especially in Asians ^30^. On the other hand, studies in an Egyptian population showed differences in VDR genotypes *Apa*-I and *Taq*-I allele distribution and frequency between diabetic individuals and controls ^31^. There are, of course, a few studies demonstrating that the *Apa*I polymorphism was related to obesity and blood glucose. Most articles focused on type 2 diabetes or polycystic ovary syndrome and other *VDR* gene polymorphisms ^21^^,^^32^^-^^36^.

Given that the outcomes depend on numerous environmental, cultural, geographical, and socioeconomic factors, the results obtained differ by population. Like other studies, ours was not without limitations. The size of the study population and the fact that not all variants in the *VDR* gene were evaluated are the most important ones. Vitamin D serum levels were not assessed either. On the other hand, the most important strengths of the study were the ethnic homogeneity of participants and the association of genotype and blood biochemical factors with BMI.

We found relationships between the *VDR* gene *Apa*I polymorphism and obesity in an Iranian population. The relation of ApaI AA genotype and A allele to obesity phenotypes can make them obesity potential predictors. These findings suggest that the *Apa*I polymorphism can predict the increased risk of obesity and help to identify novel treatment strategies for this metabolic disorder. We recommend future studies with larger samples to analyze the relationship between environmental factors and single nucleotide polymorphisms involved in obesity in different Iranian populations.

## References

[1] Walley AJ, Asher JE, Froguel P (2009). The genetic contribution to non-syndromic human obesity. Nat Rev Genet.

[2] Kopelman P (2007). Health risks associated with overweight and obesity. Obes Rev.

[3] World Health Organization W (2000). Obesity: Preventing and managing the global epidemic. WHO Technical Report Series 894.

[4] Blum M, Dolnikowski G, Seyoum E, Harris SS, Booth SL, Petersonet J (2008). Vitamin D (3) in fat tissue. Endocrine.

[5] Hypponen E, Power C (2006). Vitamin D status and glucose homeostasis in the 1958 British birth cohort: The role of obesity. Diabetes Care.

[6] Wortsman J, Matsuoka LY, Chen TC, Lu Z, Holick MF (2000). Decreased bioavailability of vitamin D in obesity. Am J Clin Nutr.

[7] Uitterlinden AG, Fang Y, van Meurs JB, Pols HA, van Leeuwen JP (2004). Genetics and biology of vitamin D receptor polymorphisms. Gene.

[8] Tabrizi JS, Sadeghi-Bazargani H, Farahbakhsh M, Nikniaz L, Nikniaz Z (2018). Prevalence and associated factors of overweight or obesity and abdominal obesity in Iranian population: A population-based study of Northwestern Iran. Iran J Public Health.

[9] Mirzazadeh A, Sadeghirad B, Haghdoost AA, Bahreini F, Rezazadeh Kermani M (2009). The prevalence of obesity in Iran in recent decade; a systematic review and meta-analysis study. Iran J Public Health.

[10] Faghfouri AH, Faghfuri E, Maleki V, Payahoo L, Balmoral A, Khaje Bishak Y (2020). A comprehensive insight into the potential roles of VDR gene polymorphism in obesity: A systematic review. Arch Physiol Biochem.

[11] Vasilopoulos Y, Sarafidou T, Kotsa K, Papadimitriou M, Goutzelas Y, Stamatis C (2013). VDR Taql is associated with obesity in the Greek population. Gene.

[12] Chen X, Wang W, Wang Y, Han X, Gao L (2019). Vitamin D receptor polymorphisms associated with susceptibility to obesity: A meta-analysis. Med Sci Monit.

[13] World Health Organization (2013). Obesity and overweight.

[14] Meamar R, Javadirad SM, Chitsaz N, Asadian M, Kazemi M, Ostadsharif M (2017). Vitamin D receptor gene variants in Parkinson’s disease patients. Egypt J Med Hum Genet.

[15] Khammissa RA, Fourie J, Motswaledi MH, Ballyram R, Lemmer J, Feller L (2018). The biological activities of vitamin D and its receptor in relation to calcium and bone homeostasis, cancer, immune and cardiovascular systems, skin biology, and oral health.

[16] Fleet JC (2017). The role of vitamin D in the endocrinology controlling calcium homeostasis. Mol Cell Endocrinol.

[17] Bid HK, Mishra DK, Mittal RD (2005). Vitamin-D receptor (VDR) gene (Fok-I, Taq-I and Apa-I) polymorphisms in healthy individuals from north Indian population. Asian Pac J Cancer Prev.

[18] Zmuda JM, Cauley JA, Ferrell RE (2000). Molecular epidemiology of vitamin D receptor gene variants. Epidemiol Rev.

[19] Ogunkolade BW, Boucher BJ, Prahl JM, Bustin SA, Burrin JM, Noonan K (2002). Vitamin D receptor (VDR) mRNA and VDR protein levels in relation to vitamin D status, insulin secretory capacity, and VDR genotype in Bangladeshi Asians. Diabetes.

[20] Al-Daghri NM, Guerini FR, Al-Attas OS, Alokail MS, Alkharfy KM (2014). Vitamin D receptor gene polymorphisms are associated with obesity and inflammosome activity. PLoS ONE.

[21] Rahmadhani R, Zaharan NL, Mohamed Z, Moy FM, Jalaludin MY (2017). The associations between VDR BsmI polymorphisms and risk of vitamin D deficiency, obesity and insulin resistance in adolescents residing in a tropical country. PLoS ONE.

[22] Ferrarezi DAF, Bellili-Munoz N, Nicolau C, Cheurfa N, Guazzelli IC, Frazzatto E (2012). Allelic variations in the vitamin D receptor gene, insulin secretion and parents’ heights are independently associated with height in obese children and adolescents. Metabolism.

[23] Correa-Rodríguez M, Carrillo-Ávila JA, Schmidt-RioValle J, González-Jiménez E, Vargas S, Martín J (2018). Genetic association analysis of vitamin D receptor gene polymorphisms and obesity-related phenotypes. Gene.

[24] Al-Hazmi AS, Al-Mehmadi MM, Al-Bogami SM, Shami AA, Al-Askary AA, Alomery AM (2017). Vitamin D receptor gene polymorphisms as a risk factor for obesity in Saudi men. Electronic Physician.

[25] Ostadsharif M, Rashidi-Khorasgani F (2015). The relationship of FokI polymorphism in vitamin D receptor (VDR) gene and obesity. J Isfahan Med Sch.

[26] Jakubowska-Pietkiewicz E, Klich I, Fendler W, Mlynarski W, Chlebna-Sokol D (2013). Effect of vitamin D receptor gene (VDR) polymorphism on body height in children-own experience. Postepy Hig Med Dosw (Online).

[27] Nosratabadi R, Arababadi MK, Salehabad VA, Shamsizadeh A, Mahmoodi M, Sayadi AR (2010). Polymorphisms within exon 9 but not intron 8 of the vitamin D receptor are associated with the nephropathic complication of type-2 diabetes. Int J Immunogenet.

[28] Tokita A, Matsumoto H, Morrison NA, Tawa T, Miura Y, Fukamauchi K (1996). Vitamin D receptor alleles, bone mineral density and turnover in premenopausal Japanese women. J Bone Miner Res.

[29] Fountas L, Moutsatsou P, Kastanias I, Tamouridis N, Tzanela M, Anapliotou M (1999). The contribution of vitamin d receptor gene polymorphisms in osteoporosis and familial osteoporosis. Osteoporos Int.

[30] Li L, Wu B, Liu J-Y, Yang L-B (2013). Vitamin D receptor gene polymorphisms and type 2 diabetes: A meta-analysis. Arch Med Res.

[31] Kamel MM, Fouad SA, Salaheldin O, Abd El-Razek AA, Abd El-Fatah AI (2014). Impact of vitamin D receptor gene polymorphisms in pathogenesis of type-1 diabetes mellitus. Int J Clin Exp Med.

[32] Fatma H, Abdul SN (2019). Association of vVitamin D receptor gene BsmI polymorphism with type 2 diabetes mellitus in Pakistani population. Afri Health Sci.

[33] Sikander M (2017). Relationship of ApaI VDR gene polymorphism to type 2 diabetes mellitus in Pakistani population. Electronic J Biol.

[34] Santos BR, Lecke SB, Spritzer PM (2018). Apa-I polymorphism in VDR gene is related to metabolic syndrome in polycystic ovary syndrome: A cross-sectional study. Reprod Biol Endocrinol.

[35] Angel B, Lera L, Márquez C, Albala C (2018). The association of VDR polymorphisms and type 2 diabetes in older people living in community in Santiago de Chile. Nutr Diabetes.

[36] Mohamed S, El-Askary A (2017). Vitamin D receptor gene polymorphism among Egyptian obese children. Asian J Clin Nutr.

